# On the Origins of Signal Variance in FMRI of the Human Midbrain at High Field

**DOI:** 10.1371/journal.pone.0062708

**Published:** 2013-04-26

**Authors:** Robert L. Barry, Mariam Coaster, Baxter P. Rogers, Allen T. Newton, Jay Moore, Adam W. Anderson, David H. Zald, John C. Gore

**Affiliations:** 1 Vanderbilt University Institute of Imaging Science, Nashville, Tennessee, United States of America; 2 Department of Radiology and Radiological Sciences, Vanderbilt University Medical Center, Nashville, Tennessee, United States of America; 3 Neuroscience Graduate Program, Vanderbilt University, Nashville, Tennessee, United States of America; 4 Department of Biomedical Engineering, Vanderbilt University, Nashville, Tennessee, United States of America; 5 Departments of Psychology and Psychiatry, Vanderbilt University, Nashville, Tennessee, United States of America; University of Minnesota, United States of America

## Abstract

Functional Magnetic Resonance Imaging (fMRI) in the midbrain at 7 Tesla suffers from unexpectedly low temporal signal to noise ratio (TSNR) compared to other brain regions. Various methodologies were used in this study to quantitatively identify causes of the noise and signal differences in midbrain fMRI data. The influence of physiological noise sources was examined using RETROICOR, phase regression analysis, and power spectral analyses of contributions in the respiratory and cardiac frequency ranges. The impact of between-shot phase shifts in 3-D multi-shot sequences was tested using a one-dimensional (1-D) phase navigator approach. Additionally, the effects of shared noise influences between regions that were temporally, but not functionally, correlated with the midbrain (adjacent white matter and anterior cerebellum) were investigated via analyses with regressors of ‘no interest’. These attempts to reduce noise did not improve the overall TSNR in the midbrain. In addition, the steady state signal and noise were measured in the midbrain and the visual cortex for resting state data. We observed comparable steady state signals from both the midbrain and the cortex. However, the noise was 2–3 times higher in the midbrain relative to the cortex, confirming that the low TSNR in the midbrain was not due to low signal but rather a result of large signal variance. These temporal variations did not behave as known physiological or other noise sources, and were not mitigated by conventional strategies. Upon further investigation, resting state functional connectivity analysis in the midbrain showed strong intrinsic fluctuations between homologous midbrain regions. These data suggest that the low TSNR in the midbrain may originate from larger signal fluctuations arising from functional connectivity compared to cortex, rather than simply reflecting physiological noise.

## Introduction

The midbrain dopamine (DA) neurotransmitter system is housed primarily within the ventral tegmental area (VTA) and the substantia nigra pars compacta (SN). DA neurons from these two regions are known to have complex interconnections with cortical and sub-cortical brain areas, and they posses unique firing properties [1] known to be associated with various goal-directed behaviors [2]–[6]. DA circuitry dysfunction is also implicated in various neuropsychiatric illnesses including Parkinsons [7], schizophrenia [8], and drug addiction [9].

Functional MRI at 7 Tesla (7 T) is particularly promising for studying the midbrain because acquisitions at higher field strengths should show higher blood oxygenation level dependent (BOLD) contrast, which may allow higher resolution functional imaging [10]–[13]. Anatomic studies of the midbrain have already demonstrated the advantages of ultra-high field imaging [14]–[17], revealing structural details with close concordance to histology. However, the functional profile of the midbrain VTA and SN in humans and in disease states is yet to be fully elucidated.

Standard single-shot 2-D gradient echo Echo Planar Imaging (EPI) sequences have been conventionally used for acquiring fMRI data on 1.5 T and 3 T scanners [18], [19]. This fast imaging sequence allows for full brain multi-slice coverage with acquisition times of a few seconds, and has been used reliably to assess functional signal changes both cortically and subcortically at lower field strengths. However, at high field strengths, there are drawbacks to using this approach, especially within the brainstem. Gradient echo EPI imaging sequences suffer from susceptibility artifacts, distortion, and signal drop out at the interfaces of bone, tissue and air. Even with the use of parallel imaging techniques (SENSitivity Encoding, SENSE [20]), 2-D EPI is unable to reliably acquire functional imaging data in areas with strong magnetic field gradients. Additionally, slice-selective 2-D data acquisition allows SENSE to be implemented only in one phase encoding direction.

Fast 3-D gradient echo acquisitions have been shown to reduce distortions from field variations and can therefore be an effective alternative for imaging the midbrain regions [21]–[23]. The Fast Field Echo (FFE) sequence acquires data in 3-D, with two phase encoding directions that can both be accelerated with SENSE, allowing complete volumes to be acquired at high temporal resolutions (1–2 s per volume). This type of scan produces images that are less distorted because the time spent traversing k-space during any given shot is reduced. Moreover, this type of scan is less susceptible to physiological noise [24]. A related 3-D pulse sequence that may also be used for functional imaging at ultra-high field is PRESTO (PRinciples of Echo Shifting using a Train of Observations) [25], [26]. In this multi-shot 3-D volume acquisition, an echo shifting technique is implemented to make optimal use of the time between the radio frequency excitation pulse and the readout by applying the next excitation before signal readout [27], [28]. This 3-D T2*-weighted MR sequence has previously been used to record BOLD responses from the cortex at 7 T [29]. The spatial resolution in 3-D multi-shot FFE can be higher than 2-D EPI because higher spatial frequencies can be sampled shortly after RF excitation. However, a drawback of using multi-shot sequences is higher sensitivity to between-shot motion and phase errors [30]. The use of phase correction algorithms with navigator echoes [31], [32] may help to mitigate some of these errors.

There are particular challenges to imaging the midbrain in comparison to the cortex at 7 T. Higher field strength imaging of the brainstem may suffer image distortions caused by macroscopic magnetic susceptibility variations in the brain and signal changes related to the behavior of radiofrequency fields at high frequencies. The midbrain is located near the interpeduncular fossa (IPF), which is an area of cerebral spinal fluid (CSF) flow. Magnetic susceptibility varies within and across these tissue boundaries, thereby distorting the applied magnetic field. This results in signal artifacts that include geometric distortions (macroscopic spatial image distortion) and variations in signal intensity (due to dephasing of transverse magnetization). Moreover, since the imaging plane typically covers the auditory canal, signal drop out and various ghosting artifacts can also be observed near the midbrain. All these factors affect the spatial uniformity and temporal stability of acquired fMRI images. In general, the physiological noise caused by respiration and cardiac pulsations also increases with increasing field strength [13]. While fMRI at 7 T has been shown to be able to produce BOLD activation maps of the cortex at higher spatial resolution and/or contrast to noise ratio [29], in practice the TSNR in the midbrain regions is found to be relatively low. This lower TSNR then reduces the ability to detect task-related BOLD changes [33].

The goal of the present study was to examine the causes of the low TSNR in the midbrain and evaluate strategies for improvements. Techniques spanning the data acquisition, preprocessing and analysis stages were explored. We quantified the contributions of known physiological noise sources, of errors produced by shot-to-shot phase instabilities in 3-D multi-shot sequences, and evaluated the shared variance with regions that were temporally correlated with the midbrain. We also compared the average signal magnitudes of the midbrain and visual cortex. Additionally, we explored the functional connectivity of the VTA and SN with each other in order to determine the level of temporal covariation in the midbrain. If there is a high level of temporal covariation between midbrain areas that is not explainable by previously identified sources of noise, it could suggest that some of the intrinsic signal variability in the midbrain is of neural origin rather than reflecting random noise or quasiperiodic physiological noise.

## Technical Background

### Sources of Signal Variance in the Midbrain

There are various potential causes of temporal signal variations in the midbrain including partial volume effects, motion of CSF flow in the IPF, motion of the brainstem due to cardiac pulsatility in the arteries within and surrounding the midbrain, changes in B_0_ uniformity caused by chest movement induced by respiration, and thermal noise associated with scanner electronics [34]–[37]. In addition to the contribution of cardiac and respiratory cycles, physiological noise is also thought to include a BOLD component arising from hemodynamic and metabolic fluctuations in gray matter [38], [39]. The ratio of physiological noise to thermal noise typically increases with increasing field strength, but increasing spatial resolution at high field strengths will reduce this ratio [13].

### Techniques for Assessing and Mediating Noise

We evaluated the efficacy of several methodologies for mitigating typical sources of noise in midbrain fMRI data. These methods are briefly introduced in the following section.

#### RETROICOR

Glover and colleagues [40] demonstrated the viability of using a retrospective correction algorithm, RETROICOR, for removing periodic respiration and cardiac effects in EPI data. This method works effectively when the cardiac and respiratory temporal variations are distinct from any task-related variance. The midbrain and brainstem may be subject to cardiac pulsatile effects in particular, so functional imaging in the midbrain may benefit from the use of RETROICOR.

#### Phase regression analysis

Phase regression suppresses temporally correlated susceptibility changes that are present in both magnitude and phase data [41], [42]. Such changes may result from microscopic susceptibility effects within a voxel or macroscopic susceptibility effects due to bulk shifts in B_0_ induced by processes such as normal subject respiration. With respect to microscopic effects, voxels containing blood vessels larger than the smallest intra-cortical veins are considered to contain oriented vessels, producing changes in both the signal magnitude and phase during neural activation [42]. In comparison, smaller vessels in capillary beds are typically randomly oriented and produce predominantly magnitude changes. The fraction of BOLD signal that arises from oriented vessels is thus effectively removed by measuring and regressing (on a per-voxel basis) the influence of the phase angle from large vessels in the complex valued fMRI dataset [42]. This algorithm can also suppress the influence of extraneous phase changes associated with macroscopic shifts in magnetic susceptibility, thereby potentially increasing the detectability of genuine BOLD signal changes in the capillary bed [41].

The midbrain is highly vascularized, with vessel sizes ranging from very small venules and arterioles up to approximately 100 µm in radius, and they perfuse the medio-lateral and dorso-ventral extent of the midbrain [43]. In cross sectional views of the midbrain, the vessels supplying the SN and VTA take on a geometric orientation with the arteries and veins (both parallel in their alignment to each other) running orthogonal to the SN and VTA. These arteries and veins belong to the internal anterolateral group of mesencephalic vessels. The suitability of the phase regression algorithm for reducing noise in the midbrain has to be assessed taking into consideration the oriented vasculature in the SN and VTA.

#### Motion of the brainstem

The brainstem is surrounded by arteries and areas of CSF flow, so bulk pulsatile motions in these regions are a potential concern. Previous studies have reported brainstem displacements in the inferior and anterior directions during cardiac systole [44]. Using different prospective and retrospective cardiac-gated MR scans, previous studies have shown a rostro-caudal displacement in the midbrain-pons region of 0.16±0.2 mm with peak velocities of 1.1–1.5 mm/s [37], [45]. Another report described brainstem motion as a single rostro-caudal displacement in systole followed by a slow return to the original position in diastole [46]. Displacement according to this study involved the midbrain and brainstem descending towards the foramen magnum with velocities increasing with proximity to the foramen (<2 mm/s) and an associated medial compression of the thalami on the third ventricle (<1.5 mm/s). Quantification of the motion and displacement of the midbrain over the cardiac cycle may provide insight into the origin of BOLD signal variations. Motion can be quantified using phase contrast velocity encoding.

#### Power spectrum maps of the midbrain

Cardiac pulsatility and dynamic changes caused by respiration are known to introduce temporal instability in functional imaging data and reduce BOLD signal detectability [47], [48]. Integrating single voxel power spectra within the cardiac and respiratory frequency ranges quantify their contributions to the total variance, with the understanding that some aliasing of higher frequency cardiac power will occur. Using a very high temporal resolution scan (450 ms), data can be sampled fast enough to quantify the power in the cardiac (around 1 Hz) and respiratory (around 0.25 Hz) frequency ranges. From these data, spatial power spectrum maps can be created from the cardiac and respiratory frequency ranges to demonstrate the spatial distribution of power in the midbrain. These maps then allow for the estimation of the percentages of noise in the cardiac and respiratory frequency bands relative to the total noise power.

#### 1-D navigator during image acquisition to mediate multi-shot phase errors

Multi-shot scan sequences are susceptible to shot-to-shot phase variations. For the successful reconstruction of a slice or imaging volume, each collection of k-space lines needs to be accurately combined, or else “ghosts” are observed both within each image (stationary ghosts) as well as across time (temporal ghosts). Navigator echoes have previously been used to correct for such phase changes [32], [49]–[51]. A 1-D navigator echo represents the central line of k-space before applying phase encode gradients, and thus provides an estimate of the baseline phase [32]. We use this technique in our cortical imaging studies at 7 T and observe a twofold improvement in resting state TSNR in the cortex (unpublished observations). One of the disadvantages in using this approach is the assumption that phase variations are the same across a given slice or throughout a volume in a 3-D acquisition, which in general may not be true.

#### Regressors of no interest in the GLM analysis

Krebs and colleagues [52] used the average time course from the anterior cerebellum as a regressor of no interest in analyses of superior colliculus BOLD signal at 7 T. Improvements in the task-related t-statistics were demonstrated. This method can be potentially advantageous because it makes no assumptions concerning the sources of noise in the data, and also attempts to remove all temporal variations of whatever origin that are common to both regions. To translate this approach for the midbrain, the cerebral peduncles (white matter area adjacent to the SN) and the anterior cerebellum (posterior to the superior colliculus) may be used as regressors of no interest. CSF flow in the IPF and the cerebral aqueduct, as well as pulsatility of the posterior cerebral arteries skirting the lateral edges of the midbrain, may produce signal variability in the entire midbrain region including the cerebral peduncles and the anterior cerebellum. Accounting for this signal variance in the cerebral peduncles and the anterior cerebellum should remove common noise from the VTA and SN.

#### Comparing signal magnitudes in the midbrain and cortex

In order to compare the absolute magnitudes of MR signal in the midbrain and visual cortex, relevant imaging parameters (e.g., T1, TE, and T2*) must be measured for each tissue. These values can be inserted into the appropriate steady state signal equation, which is 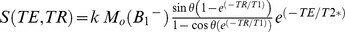 [53], where S is the steady state signal, *k* represents an overall scaling constant, M_0_ is the equilibrium magnetization, B_1_ is the receive radio frequency field, TR is the repetition time, T1 is the rate of longitudinal relaxation, TE is the echo time, T2* is the decay rate of transverse magnetization and theta (θ) is the actual flip angle (calculated by multiplying the nominal flip angle with the measured relative B_1_ amplitude in the given voxel). This equation, in combination with measured signal values, allows *k*M_0_ and then experimental values of S to be evaluated separately for the midbrain and cortex. Signals from different protocols (2-D EPI, 3-D FFE, and 3-D PRESTO) may be similarly estimated.

## Methods

### Participants

Healthy normal participants were scanned during the execution of an activation task and also in a resting sate protocol using different sequences (2-D EPI, 3-D FFE and 3-D PRESTO). All participants provided written informed consent for the study, which was approved by the institutional review board of Vanderbilt University Medical Center. Five participants (5 males, mean age = 26 years) completed the FFE study with the task, and, in this first study, only the magnitude data were recorded. For all following studies, both real and imaginary image data were retained after magnitude reconstruction. An additional five participants (3 males, mean age = 30.5 years) then completed the FFE study with task. Five participants (3 males, mean age = 31.2 years) completed the EPI study with task. Five participants (3 males, mean age = 32 years) completed the PRESTO study with task. Six participants (4 males, mean age = 35.2 years) completed the resting state fMRI scans for EPI, FFE and PRESTO sequences.

To assess the potential impact of different noise sources, additional resting state scans were performed. One participant (male, 27 years old) completed a study that collected high temporal resolution PRESTO and EPI resting state data to compute spectral power maps without aliasing. One participant (male, 27 years old) completed a resting state study that used a navigator pulse in 3-D PRESTO and 3-D FFE scans to measure the influence of multi-shot phase instabilities. One participant (male, 26 years old) completed a retrospective cardiac-gated phase contrast resting state scan for measuring brainstem motion. Two participants (male, mean age = 30.5 years) completed the resting state study that collected imaging parameters to compare signal in the midbrain and the cortex.

### fMRI Data Acquisition

Images were acquired using a 7 T Achieva Scanner (Philips Healthcare, Best, the Netherlands) with a 32-channel head coil (Nova Medical, Wilmington, MA). Whole brain 3-D anatomical scans were acquired using a T1-weighted Turbo Field Echo pulse sequence in each scan session. 138 slices were collected in the sagittal orientation with field of view (FOV) = 256 (FH)×256 (AP)×172.5 (RL) mm, parallel imaging factor (SENSE, [20]) = 2 in the AP direction, TR = 3.0 ms, TE = 1.35 ms, voxel resolution = 1.25 mm×1.25 mm×1.25 mm with zero gap, flip angle (FA) = 7^o^, acquisition time = 2 min 12 s. This high resolution structural scan was used to determine the slice placement for the functional scans.

T2*- weighted BOLD images for the sequences described below were acquired in an oblique axial orientation with slices positioned to cover the dorso-ventral extent of the midbrain from the inferior edge of the caudate head to the dorsal border of the pons. The axial sections were tilted to be parallel to a plane bisecting the mammillary body and the inferior frontal lobe. First-order shimming for the 2-D single-shot EPI scans was performed over the midbrain area [54]. A third-order pencil beam shim was used for the 3-D FFE and 3-D PRESTO scans over the midbrain area [55], [56]. For the task-based runs, the onset of each run was triggered from the scanner such that fMRI data collection and stimulus presentation were synchronized. Three runs were collected for each task-based scan. Table 1 lists the imaging parameters for the task-based fMRI runs. The resting state scans had the same parameters as the task scans, except with fewer volumes (200 dynamics after reaching steady-state magnetization). Two resting state runs were collected for each sequence type in every individual. The duration of each EPI scan was 7 min 4 s, the duration of each FFE scan was 7 min 1 s, and the duration of each PRESTO scan was 7 min 1 s.

**Table 1:** Imaging parameters for task-based 2-D EPI, 3-D PRESTO and 3-D FFE fMRI sequences.

Sequence	2D EPI	3D FFE	3D PRESTO
**FOV (mm)**	220 (AP)×190 (RL)×23.94 (FH)	170 (AP)×170 (RL)×21.28 (FH)	240 (AP)×220 (RL)×100 (FH)
**Voxel resolution (mm^3^)**	1.96×2.03×2.00	1.33×1.33×1.33	2.50×2.56×2.50
**Volume acquisition time (ms)**	2000	2000	2000
**TR (ms)**	2000	25	18
**TE (ms)**	24	17	26
**# Slices**	18	16	40
**SENSE**	3 (RL)	3.1 (RL)	2.4 (RL), 1.5 (FH)
**Flip Angle (deg)**	75	15	10
**# Dynamics**	372	372	372
**Scan Time (min:sec)**	12∶48	12∶50	12∶45

FOV is the field of view, TR is the repetition time, TE is the echo time and SENSE is the acceleration factor in the Right-Left (RL) and Anterior-Posterior (AP) planes.

#### Resting state scans with high temporal resolution and navigator pulse

To characterize noise in the cardiac and respiratory frequencies, we collected high temporal resolution resting state scans using EPI and PRESTO sequences. For the multi-shot PRESTO scans, a 1-D navigator was implemented to test differences between TSNR before and after shot-to-shot phase error correction. We also applied the navigator with a low temporal resolution FFE scan to compare results with the high temporal resolution scan. See Table 2 for a complete list of imaging parameters.

**Table 2:** Imaging parameters for 2-D EPI, 3-D PRESTO and 3-D FFE fMRI sequences with high temporal resolution and navigator pulse.

Type of Scan	High *temp. res.*	High *temp. res*. & no *nav.* pulse	with *nav.* pulse	no *nav.* pulse	with nav. pulse
**Sequence**	2D EPI	3D PRESTO	3D PRESTO	3D FFE	3D FFE
**FOV (mm)**	220 (AP)×172.86 (RL)×8 (FH)	170 (AP)×170 (RL)×16 (FH)	170 (AP)×170 (RL)×12 (FH)	170 (AP)×170 (RL)×21.28 (FH)	170 (AP)×170 (RL)×21.28 (FH)
**Voxel resolution (mm^3^)**	1.96×2.03×2.00	2.02×2.07×2.00	2.00×2.00×2.00	1.33×1.33×1.33	1.33×1.33×1.33
**Volume acquisition time (ms)**	423	346	360	2000	2000
**TR (ms)**	423	12	15	25	25
**TE (ms)**	20	19	22	17	17
**# Slices**	4	8	6	16	16
**SENSE**	3 (RL)	2 (AP)	2 (AP)	3.1 (RL)	3.1 (RL)
**Flip Angle (deg)**	28	10	10	15	15
**# Dynamics**	200	265	265	150	150
**Scan Time (min:sec)**	1∶39	1∶43	1∶47	5∶5	5∶6

FOV is the field of view, TR is the repetition time, TE is the echo time and SENSE is the acceleration factor in the Right-Left (RL) and Anterior-Posterior (AP) planes.

#### Retrospective cardiac gating for brainstem motion

Retrospective cardiac-gated phase contrast scans were used to evaluate the extent of brainstem motion in the anterior-posterior (AP), right-left (RL) and superior-inferior (Head-Foot, HF) directions. A finger pulse monitor was used to synchronize the scans with the cardiac cycle. The cardiac cycle was retrospectively divided into 16 intervals. Over the course of the scan, imaging data from 80–90 heart beats were acquired. The imaging parameters for this 2-D scan were: FOV = 220 mm (AP)×180 mm (RL)×5 mm (FH), SENSE factor = 2 in the RL direction, TR = 18 ms, TE = 13 ms, one slice with a voxel resolution = 1.00 mm×0.99 mm×5.00 mm, FA = 10^o^ and number of signal acquisitions (NEX) = 2. Quantitative phase contrast measurements were derived using a velocity encoding factor of 1 cm/s (range of phase shift<π). Images were acquired in each of the AP, RL and HF directions, each run lasting 1 min 35 s.

#### Resting state scans comparing midbrain and visual cortex

To compare the signals in the midbrain and cortex, a series of resting state scans were implemented in an oblique axial orientation with slices positioned to cover the dorso-ventral extent of the midbrain from the inferior edge of the caudate head to the dorsal border of the pons. The axial sections were tilted to make them parallel to a plane bisecting the mammillary body and the inferior occipital lobe. See Table 3 for a complete list of imaging parameters.

**Table 3:** Imaging parameters for 2-D EPI, 3-D PRESTO and 3-D FFE fMRI sequences for comparing signal and noise in the midbrain and cortex.

Sequence	2D EPI	3D PRESTO	3D FFE
**FOV (mm)**	220 (AP)×172.86 (RL)×17.6 (FH)	223.46 (AP)×173 (RL)×17.6 (FH)	170 (AP)×170 (RL)×21.28 (FH)
**Voxel resolution (mm^3^)**	1.96×2.03×2.00	1.97×2.58×2.20	1.33×1.33×1.33
**Volume acquisition time (ms)**	446	440	2000
**TR (ms)**	446	16	26
**TE (ms)**	18	24	17
**# Slices**	8	8	16
**SENSE**	3 (RL)	2 (AP), 1.3 FH	3.1 (RL)
**Flip Angle (deg)**	28	10	15
**# Dynamics**	200	265	150
**Scan Time (min:sec)**	1∶39	1∶43	5∶29

FOV is the field of view, TR is the repetition time, TE is the echo time and SENSE is the acceleration factor in the Right-Left (RL) and Anterior-Posterior (AP) planes.

#### Resting state scans to estimate signal magnitudes in midbrain and cortex

A multi-echo (10 echoes) 3-D FFE scan was performed to construct a T2* map. The imaging parameters for this scan included: FOV = 240 (AP)×199.2 (RL)×26.4 (FH) mm, SENSE factor = 2 in the RL direction and 1.2 in the FH direction, TR (time between shots) = 45 ms, TE_1_ = 4.6 ms, ΔTE = 3 ms, voxel resolution = 0.6 mm×0.6 mm×1.20 mm, 22 slices and FA = 10^o^. The scan duration was 3 min 16 s. After data acquisition, the T2* map was calculated by performing a voxel-by-voxel least-squares exponential fit to the multi-echo signal.

To determine the spatial distribution of the radiofrequency field (B_1_), a map of relative field amplitude was created using a series of 11 single-slice gradient recalled echo images acquired at flip angles ranging from 10^o^ to 210^o^ in 20 degree increments [57], with TR and TE, respectively, set to 6000 ms and 6.8 ms. The B_1_ maps scans had the following imaging parameters: FOV = 240 (AP)×192 (RL)×147 (FH) mm, voxel resolution = 3.12 mm×3 mm×3 mm, and 49 slices. The duration for each scan was 7 min 54 s. The B_1_ values at each voxel were calculated using a standard least-squares fitting method [57].

A B_0_ map was also collected to allow distortion correction of the images used for the B_1_ map (since those scans were EPI). The B_0_ map was acquired with the following imaging parameters: FOV = 240 (AP)×192 (RL)×165 (FH) mm, no SENSE acceleration, TR = 4.1 ms, TE = 1.63 ms, ΔTE = 1 ms, voxel resolution = 3 mm×3 mm×3 mm, 55 slices and FA = 10^o^. The scan duration was 36 s.

A T1 map was made using an inversion recovery sequence. Each inversion recovery scan had a different inversion delay (TI) prior to signal acquisition (7 time points: 100 ms, 200 ms, 400 ms, 800 ms, 1600 ms, 3200 ms, and 5000 ms). The inversion recovery scans had the following imaging parameters: FOV = 240 (AP)×192 (RL)×26 (FH) mm, TR = 8000 ms, voxel resolution = 1.5 mm×1.5 mm×2 mm, and 9 slices. The scan duration was 3 min 44 s for each scan. The time series from these scans were fit using the inversion recovery signal equation: 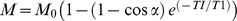 where M_0_, α (the flip angle of the inversion pulse), and T1 are the fit parameters.

A T2-weighted GRASE scan was also performed at the same resolution as the T1 maps to aid in drawing ROI masks. The T1 maps themselves did not have enough contrast to delineate between structures of interest in the midbrain.

### Physiological Monitoring

The participant’s pulse rate was monitored using a finger peripheral pulse unit (PPU) that interfaced with the scanner. It was placed on the left middle or index finger of each participant. The participant’s respiration was monitored using a respiratory belt placed around the abdomen or sternum area. The location of the belt placement was chosen based on the region that displayed largest motion during normal breathing. Data from both the PPU and respiration were sampled at 100 Hz and stored in a log file after each run.

### Task Based Experimental Design

During activation scans, subjects performed the Monetary Incentive Delay (MID) task (details of which have been previously published [58]). We included a prediction error component to this task since the midbrain DA neurons have been shown to be sensitive to the timing and magnitude of the expected reward. Slow event related stimulation paradigm design details can be found in Coaster, 2012 [33] (Dissertation thesis chapter V).

### fMRI Data Analysis

Functional images were analyzed using Statistical Parametric Mapping (SPM5; Wellcome Department of Imaging Neuroscience, University College, London, UK). Slice time correction, motion correction, and RETROICOR were applied to all 2-D EPI data. In the 3-D FFE and PRESTO multi-shot sequences, slice time correction was not performed because data from the whole volume are collected simultaneously. RETROICOR was employed using in house software based on Glover and colleague’s [40] original algorithm implemented in Matlab (version R2010a, MathWorks, Natick, MA).

For the fMRI scans in which the MID task was incorporated, a general linear regression model (GLM) was used to analyze the data. Each participant’s data were inspected for excessive motion and only subjects with <1.5 mm motion in every direction across all runs were included in the analyses.

### Data Analysis Techniques

#### Estimating TSNR

Three dimensional region of interest (ROI) masks were drawn on the functional datasets for the VTA and SN, localizing their boundaries with respect to neighboring regions as predicted by atlases [43], [59]. The mean signal for each run and the residual variance were extracted from each of the ROI masks. To calculate the TSNR in the ROI, the mean signal was divided by the square root of the temporal variance. For the resting state scans, we calculated the mean and standard deviation (SD) in every voxel over time. TSNR was then calculated on a per-voxel basis by dividing the mean signal by the SD calculated across all time points. The VTA and SN masks were used to extract the TSNR in each ROI by taking the mean of the TSNR in the voxels falling within the ROI.

#### Phase regression analysis

A rigid body motion correction was implemented on the reconstructed magnitude data to estimate within-plane translation and rotation parameters. The transformation matrix for each dynamic was then applied to the original real and imaginary data. These motion-corrected real and imaginary data were then combined to produce new motion corrected magnitude and phase image. Linear, quadratic and cubic polynomial detrending was applied to motion corrected magnitude and phase data. This was followed by temporal phase unwrapping and phase regression analysis on a voxel-wise basis to remove the influence of BOLD effects produced by large vessels and spatiotemporally varying magnetic field inhomogeneities [41], [42].

#### Evaluating motion of the brainstem using retrospective gating

An arbitrary ROI placed in fatty tissue surrounding the skull was chosen as a comparison region that is expected to move little over the cardiac cycles. The entire midbrain region, VTA, and SN were drawn on the magnitude image. The masks of the midbrain ROIs (drawn on the magnitude data) were used to extract velocity information from the phase data. We used the difference in velocity between midbrain ROIs and the fatty tissue ROI to estimate the relative velocity of the midbrain ROIs over a cardiac cycle. Using the velocity information and the time interval of each phase, we calculated the net displacement from the original position as well as average and peak velocities.

#### Power spectrum maps in the cardiac and frequency ranges

From the high temporal resolution PRESTO and EPI sequences, we were able to sample the data fast enough to capture the cardiac and respiratory frequencies. For all the imaging slices acquired, we calculated spatial power spectra maps in the cardiac (0.8–1.1 Hz) and respiratory (0.08–0.25 Hz) frequency ranges separately to estimate the percent contribution to the total variance within the cardiac and respiratory frequency ranges.

#### 1-D navigator to correct for multi-shot phase errors

A navigator echo was collected before applying the phase encode gradients [32]. During the reconstruction stage, the phase of the navigator echo was subtracted from the phase for all lines of k-space (adjusting for the time at which each line was acquired). This was applied for the resting state high temporal resolution PRESTO and low temporal resolution FFE sequences.

#### Implementing a regressor of no interest

Task-based FFE data from the first five participants were used in this analysis. 3-D ROIs in the cerebral peduncles (white matter, WM) located adjacent to the SN and the anterior cerebellum (ACb) posterior to the superior colliculus were identified as regions where no task-based activations would occur. We extracted the average time course in each of these regions (method described in [52]) and performed a slice-by-slice Pearson correlation analysis between the average time course in the WM, the ACb and the average time course in the VTA as well as the SN. Both the left and right WM average time courses were included as regressors of no interest along with the other MID task regressors for the full brain GLM SPM analysis. A separate analysis was performed in a similar way using the left and right ACb ROIs. These five FFE datasets were motion corrected and included in the regressor of no interest analysis with and without RETROICOR.

#### Time course correlations in resting state data

The resting state data were band-pass filtered in FSL using the FEAT analysis toolbox (Analysis Group, FMRIB, Oxford, UK [60], [61], after initial preprocessing in SPM5). This temporal filtering retained data with frequencies between 0.01–0.1 Hz. 3-D ROI masks were drawn for the VTA, SN and WM (cerebral peduncles) midbrain regions in each resting state scan. These masks were used to extract the average time course in each ROI from the functional dataset. Pearson correlations between the time courses of different ROI pairs were then performed in SPSS (IBM SPSS Inc. release 19). Additionally, partial correlations were performed between the SN and VTA accounting for the shared variance with the neighboring midbrain WM. This analysis allowed determination of correlations specific to the midbrain DA regions after excluding global effects that also influence the neighboring WM. A repeated measures ANOVA was implemented using SPSS (IBM SPSS Inc. release 19) on the filtered partial correlation results. Statistical results without correction for multiple comparisons are reported in this paper as they yielded similar results as with correction.

#### Functional connectivity maps

The band-pass filtered data were entered into SPM where a seed-based functional connectivity analysis was implemented using a GLM. The mean time course of an ROI was used as the regressor in the GLM analysis. Six different ROIs were selected for this analysis for each participant. These ROIs included left SN, right SN, left VTA, right VTA, left WM, and right WM. The 3-D FFE and 3-D PRESTO resting state data were used for this analysis, and each run was modeled separately.

In a separate analysis, the mean time courses of the midbrain ROIs as well as the WM were included in the GLM analysis to control for shared variance between the WM and the other ROIs. Each participant’s data were individually examined using an uncorrected voxel level threshold of p<10^−6^ (minimum cluster size = 30 voxels) for the FFE scans and an uncorrected voxel level threshold of p<10^−10^ (minimum cluster size = 30 voxels) for the PRESTO scans. These thresholds were chosen to minimize false positives and to observe robust clusters of connectivity maps.

## Results

**Figure 1:**
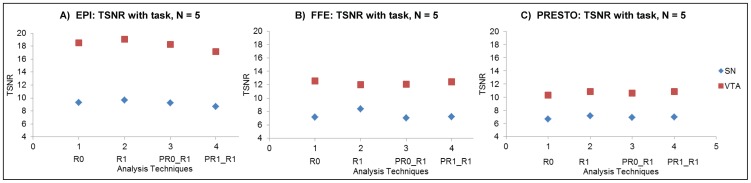
TSNR in EPI, FFE and PRESTO sequences comparing different analysis methods in task-based scans. Panel **A** 2-D EPI, panel **B**, 3-D FFE and panel **C**, 3-D PRESTO. Analysis methods include: R0 = no RETROICOR correction, R1 = only RETROICOR, PR0_R1 = no phase regression but with RETROICOR, and PR1_R1 = phase regression and RETROICOR. Regions of interest in the midbrain include the Substantia Nigra (SN), and the Ventral Tegmental Area (VTA). TSNR values in both left and right sides of the ROI are averaged; N = 5 in each sequence. TSNR values are under 10 for the SN and under 20 for the VTA irrespective of the analysis technique used.

TSNR in the midbrain was consistently below a level generally considered acceptable for functional studies. It was consistently higher in the VTA than the SN, but only in the EPI sequence did the TSNR in the VTA exceed 15. The TSNR in the SN was consistently less than 10 across the EPI, FFE and PRESTO sequences (Figure 1). As a point of comparison, cortical TSNR at 7 T typically ranges between 40–100 depending on the spatial resolution and sequence type used (unpublished observations and [62]). When RETROICOR and/or phase regression was applied to the 7 T data, the midbrain SN and VTA exhibited very little improvement in TSNR (Figure 1). Both task and resting state scans yielded comparable TSNR results across sequences (only task-based results are shown in Figure 1).

**Figure 2:**
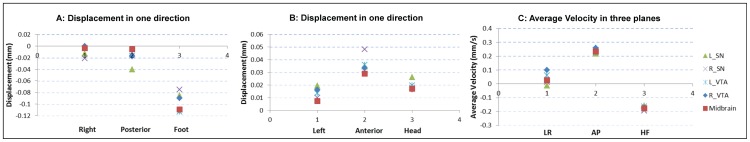
Plots of brainstem motion during cardiac systole using a retrospective cardiac gated scan. Plots display displacement and average velocity of a region of interest from the original position. These measurements are calculated along the cardiac cycle. **LR** = Left-Right direction; **AP** = Anterior-Posterior direction; **HF** = Head-Foot direction. Regions of interest include **SN** = Substantia nigra; **VTA** = Ventral Tegmental Area; L/R_SN = left/right Substantia Nigra; L/R_VTA = left/right Ventral Tegmental Area, and the **Midbrain**. Greatest displacement for the midbrain observed in the anterior and inferior directions.

Using retrospective cardiac gating, the net displacements and velocities of midbrain nuclei (SN, VTA) and the entire midbrain region were estimated in resting state scans (Figure 2). The net maximum displacement in a specific direction was highest in the foot direction (0.11 mm, Figure 2A), and second highest in the anterior direction (0.048 mm, Figure 2 B), corroborating previous findings [37], [45]. Additionally, the average velocity did not go beyond 0.19 mm/s in the head-foot direction and 0.25 mm/s in the anterior-posterior direction (Figure 2 C).

**Figure 3:**
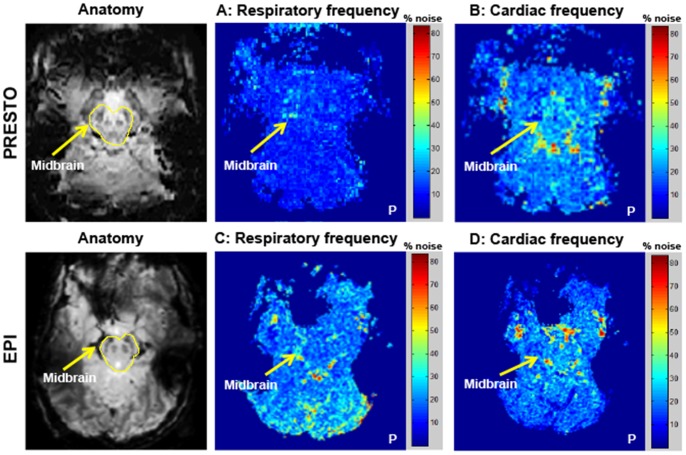
Spatial power spectrum maps of noise in midbrain areas within respiratory and cardiac frequency ranges. Power spectrum maps in the midbrain for high temporal resolution PRESTO (Panel A, B) and EPI (Panel C, D) scans sequences. Only representative slices covering the midbrain are displayed. Color scale indicates percentage of noise in respiratory and cardiac frequency ranges with respect to total noise in each voxel. Respiration frequency range = 0.08–0.25 Hz, and cardiac frequency range = 0.8–1.1 Hz. **P** denotes the posterior part of the brain. Physiological fluctuations in the midbrain (cardiac or respiratory) comprised less than half of the total noise variance in resting state data.

From the high temporal resolution PRESTO and EPI resting state data, we created power spectrum maps in the cardiac and respiratory frequency ranges (Figure 3). For PRESTO data, noise power within the cardiac frequency range revealed noise concentrated around the midbrain region, particularly in areas of CSF flow, within the IPF and areas surrounding the lateral aspects of the midbrain (Figure 3, Panel B). There was greater contribution of noise in the cardiac frequencies compared to the respiratory frequencies. Although the PRESTO sequence exhibited a high concentration of noise in the midbrain, this was not localized to the SN and VTA regions. EPI power spectrum maps revealed a different result with less concentration of noise power in the middle of the brain in both the respiratory and cardiac frequencies (Figure 3, panel C, D). Noise was not centered, however, in the midbrain regions of interest. These power spectrum maps revealed that for PRESTO data, only approximately 10% of the noise power was attributed to respiration and up to 30% to the cardiac cycle. Similarly, for EPI data, up to approximately 25% of the noise power fell within the range of normal respiration and up to 25–35% within the range of cardiac motion. These measurements suggest that the majority of noise variance in the midbrain lies in frequency ranges not typically associated with respiration and cardiac pulsatility, which was an unexpected observation because studies in the cortex have established that significant physiological noise contributions arise from both respiration and cardiac pulsatility [63], [64], [40], [65], [13], [51].

**Figure 4:**
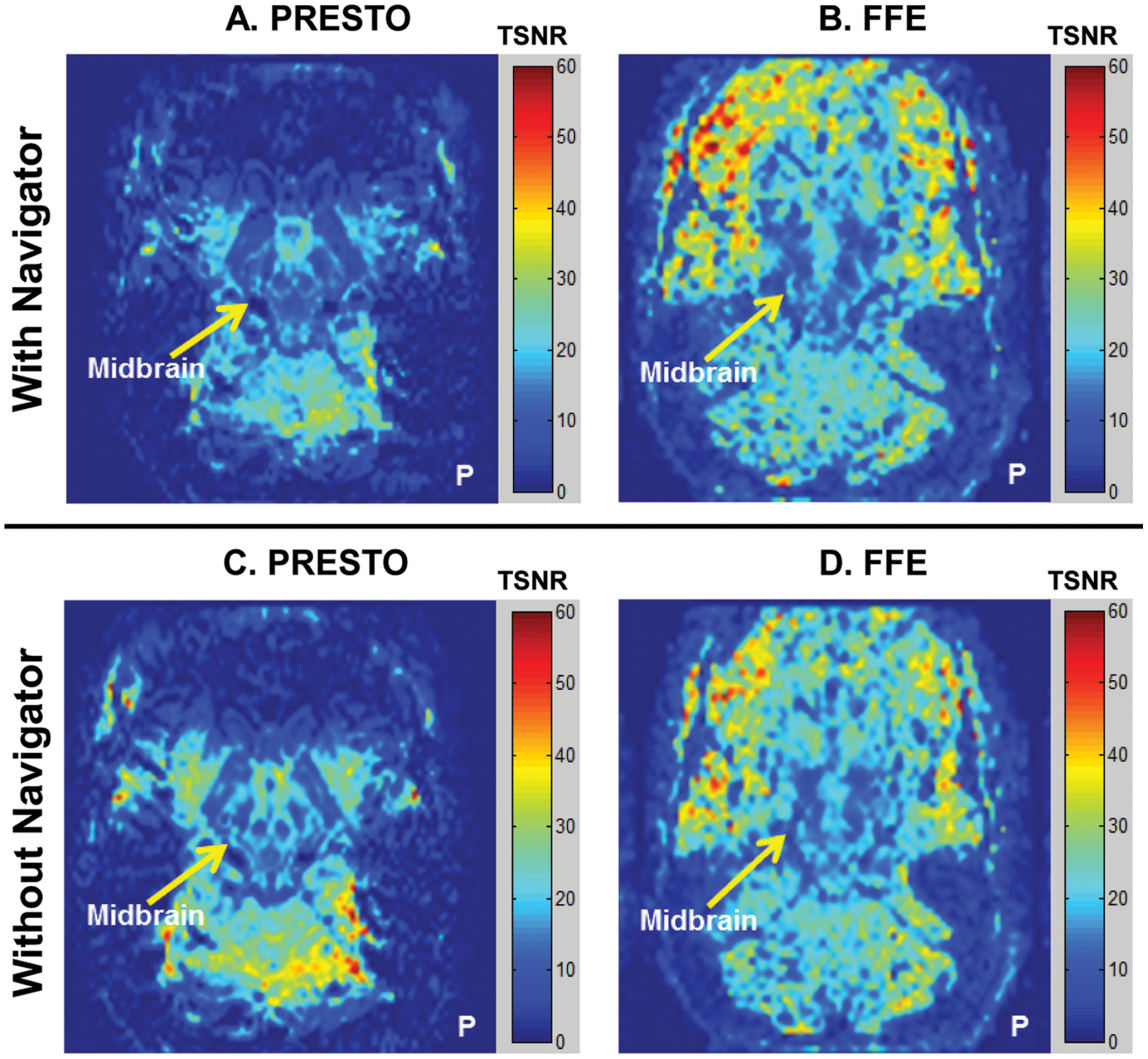
Spatial TSNR maps using a 1-D phase navigator pulse in 3-D multi-shot sequences. Only representative slices covering the midbrain are displayed. Color bar indicates TSNR values. **P** denotes the posterior part of the brain. Maps indicate a slight decrease in midbrain TSNR after 1-D navigator correction for both PRESTO and FFE data.

Using 1-D navigator correction, we observed a decrease in TSNR within the midbrain for the high temporal resolution resting state PRESTO scans (Figure 4). The TSNR, however, improved marginally in the midbrain for the low temporal resolution FFE sequence. Overall, the TSNR in the midbrain was low in both sequences (8–20 for the SN and VTA, compared to 35–50 for the cortex).

**Figure 5:**
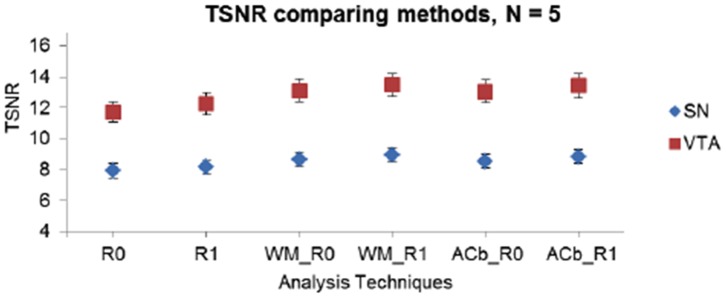
TSNR values from analysis with regressor of no interest. TSNR values in analysis with regressor of no interest using the white matter area of the cerebral peduncles (adjacent to the SN) and anterior cerebellum (posterior to the superior colliculi). The different analyses compared are: R0 = no RETROICOR, R1 = RETROICOR only, WM_R0 = WM without RETROICOR, WM_R1 = WM with RETROICOR, ACb_R0 = ACb without RETROICOR, ACb_R1 = ACb with RETROICOR. Average TSNR over five individuals in FFE data are displayed. Regions of interest in the midbrain include the Substantia Nigra (SN) and the Ventral Tegmental Area (VTA). TSNR values in both left and right sides of the ROI were averaged and N = 5. Values are under 10 for the SN and under 15 for the VTA irrespective of the analysis technique used.

The analysis with a regressor of no interest was implemented in runs with a task to regress out shared variance with regions neighboring the SN and VTA (white matter, anterior cerebellum). The average time course of the WM ROI was not highly correlated with the average time course within the VTA or SN (average Pearson correlation, SN: WM = 0.260; VTA: WM = 0.174). As a result, the fMRI data analysis with this WM regressor did not improve the t-statistics for the task-related contrasts. The TSNR was very low, ranging from 8–10 for the SN and 12–15 for the VTA (Figure 5). The time series data in the ACb (Anterior cerebellum) ROI was more highly correlated with the midbrain than the WM ROI (average Pearson correlation, SN: ACb = 0.273; VTA: WM = 0.334). Similar to the WM ROI, using the ACb ROI as a regressor of no interest in the GLM analysis did not yield a significant change in TSNR.

Within the constraints of the evaluated techniques, we did not observe an improvement in midbrain TSNR at 7 T, and the high degree of temporal variance was not readily attributed to previously identified sources of noise (physiological, phase instabilities, or temporally correlated regions). The mean signal, noise and TSNR in the SN and VTA with respect to the visual cortex revealed a notable trend across sequences. The mean signal in the cortex was approximately 1.5 times higher than in the SN (higher for FFE) and comparable to the mean signal in the VTA (slightly higher for FFE). However, the temporal standard deviation in the midbrain was approximately 1.5 (and sometimes close to 3) times higher than the noise in the cortex. As a result, resting state TSNR measured in the visual cortex using these three sequences was approximately threefold higher than in the SN and approximately twofold higher than in the VTA. Thus, while the mean signals between the cortex and midbrain were comparable, the noise was significantly higher in the midbrain compared to the cortex.

**Figure 6:**
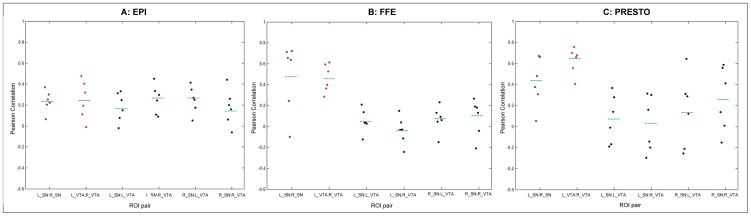
Partial correlations between midbrain ROIs in band-pass filtered fMRI data. Partial correlations between midbrain ROIs are displayed accounting for shared temporal variance with neighboring white matter in resting state filtered data (N = 6). 2-D EPI scan is shown in panel **A**, 3-D FFE in panel **B** and 3-D PRESTO is in panel **C**. Dots represent the spread of correlation values across participants. Blue dots denote the homologous left and right SN, red dots denote the homologous left and right VTA, and black dots denote the non-homologous ROI pairs. The green dashed line denotes the mean correlation across runs and across participants for each ROI pair. Statistical analysis demonstrated evidence for differences between ROI pairs for FFE (repeated measures ANOVA, F(5,25) = 8.905, p = 6×10^−5^), and PRESTO (F(5,25) = 5.601, p = 0.001), but not EPI (F(5,25) = 0.684, p = 0.640). In post-hoc tests, the differences were primarily between homologous vs. non-homologous pairings (shown in Table 4). Greater partial correlations were observed between homologous SN and VTA compared to the non-homologous pairs, particularly for the 3-D sequences.

To test whether these temporal fluctuations had a neural origin, we examined the intrinsic fluctuations (associated with functional connectivity) between midbrain ROIs. Power spectra of midbrain ROIs revealed a high degree of power in frequencies less than 0.1 Hz, intimating the possibility that low-frequency functional connectivity significantly contributed to the observed temporal fluctuations. In band-pass filtered (0.01–0.1 Hz) resting state data, partial correlations between the SN and VTA, accounting for shared variance with the WM, revealed mixed results (Figure 6). In both task and resting state 2-D EPI scans, the correlations between homologous midbrain ROIs were not different from those exhibited in the non-homologous pairs. In the 3-D FFE scans, however, partial correlations demonstrated higher correlations between the homologous pairs compared to non-homologous pairs. A similar pattern was observed for 3-D PRESTO data. The homologous pair ROI correlations were significantly higher in the resting state scan compared to the task-based scan for both 3-D sequences. We found evidence for differences between ROI pairs for FFE (repeated measures ANOVA, F(5,25) = 8.905, p = 6×10^−5^), and PRESTO (F(5,25) = 5.601, p = 0.001), but not EPI (F(5,25) = 0.684, p = 0.640). Post-hoc tests comparing connectivity measures between pairs of ROIs showed the largest difference when comparing homologous pairs to non-homologous pairs (see Table 4).

**Table 4:** Pairwise differences between homologous midbrain ROI pairs (left and right SN, left and right VTA) and their non-homologous counterparts in 2-D EPI, 3-D FFE and 3-D PRESTO data.

Sequence	Homologous	Non-homologous	p-value
EPI	L_SN:R_SN	L_SN:L_VTA	p = 0.13
EPI	L_SN:R_SN	L_SN:R_VTA	p = 0.80
EPI	L_SN:R_SN	R_SN:L_VTA	p = 0.81
EPI	L_SN:R_SN	R_SN:R_VTA	p = 0.31
EPI	L_VTA:R_VTA	L_SN:L_VTA	p = 0.23
EPI	L_VTA:R_VTA	L_SN:R_VTA	p = 0.96
EPI	L_VTA:R_VTA	R_SN:L_VTA	p = 0.98
EPI	L_VTA:R_VTA	R_SN:R_VTA	p = 0.04*
FFE	L_SN:R_SN	L_SN:L_VTA	p = 0.03*
FFE	L_SN:R_SN	L_SN:R_VTA	p = 0.02*
FFE	L_SN:R_SN	R_SN:L_VTA	p = 0.04*
FFE	L_SN:R_SN	R_SN:R_VTA	p = 0.04*
FFE	L_VTA:R_VTA	L_SN:L_VTA	p = 0.008*
FFE	L_VTA:R_VTA	L_SN:R_VTA	p = 0.005*
FFE	L_VTA:R_VTA	R_SN:L_VTA	p = 0.004*
FFE	L_VTA:R_VTA	R_SN:R_VTA	p = 0.01*
PRESTO	L_SN:R_SN	L_SN:L_VTA	p = 0.03*
PRESTO	L_SN:R_SN	L_SN:R_VTA	p = 0.05
PRESTO	L_SN:R_SN	R_SN:L_VTA	p = 0.11
PRESTO	L_SN:R_SN	R_SN:R_VTA	p = 0.26
PRESTO	L_VTA:R_VTA	L_SN:L_VTA	p = 0.009*
PRESTO	L_VTA:R_VTA	L_SN:R_VTA	p = 0.003*
PRESTO	L_VTA:R_VTA	R_SN:L_VTA	p = 0.04*
PRESTO	L_VTA:R_VTA	R_SN:R_VTA	p = 0.045*

Uncorrected p-values represent significance of differences.

* indicates significance at p<0.05.

**Figure 7:**
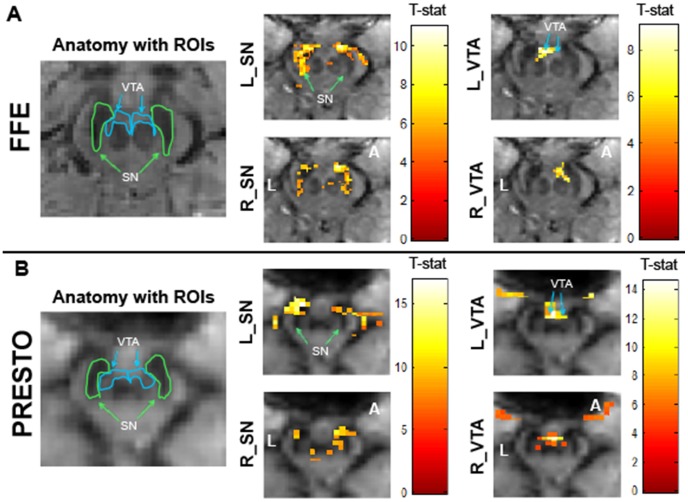
ROI seed based functional connectivity maps in the midbrain for 3-D sequences. Functional connectivity maps for FFE (Panel A) and PRESTO (Panel B) scans in a representative participant. An uncorrected voxel level threshold of p<10^−6^ (cluster size = 30 voxels) was used for the FFE scans and an uncorrected voxel level threshold of p<10^−10^ (cluster size = 30 voxels) was used for the PRESTO scans. Seed regions of interest in the midbrain include left Substantia Nigra (L_SN), right Substantia Nigra (R_SN), left Ventral Tegmental Area (L_VTA), and right Ventral Tegmental Area (R_VTA). Color bar represents T-statistic. FFE voxel size = 1.33 mm^3^ and PRESTO voxel size = 2.5 mm^3^. Axial midbrain slice sections are displayed at the level of the superior colliculus. N = 6 in each sequence type. **L** denotes the left side of the brain and **P** denotes the posterior portion of the brain. Data were motion corrected, put through RETROICOR, and band-pass filtered between 0.01 and 0.1 Hz. Robust bilateral spatial distribution of functional connectivity was observed in most ROIs.

The seed-based functional connectivity analysis exhibited spatial correlations that followed the contours of known midbrain structures (Figure 7). Both SN seeds showed strong functional connectivity with the SN in the opposite hemisphere and various parts of the striatum (pallidum, putamen). Additionally, there were associations to the dorsal prefrontal cortex and parts of the anterior cingulate. The VTA associations were primarily observed between the left and right sides. Areas reflective of arteries surrounding the midbrain and CSF flow regions proximal to the midbrain were sometimes detected in these correlation analyses, suggesting a possible residual influence from extraneous physiological fluctuations even after band-pass filtering.

## Discussion

The primary purpose of this study was to better understand the source of low TSNR in midbrain fMRI data at 7 T and to evaluate possible methods for improvement. Several techniques were implemented to characterize and correct for the high level of noise in the midbrain, but none were successful in mitigating the noise and increasing the TSNR. We therefore also explored whether intrinsic fluctuations within midbrain regions could account for the low TSNR.

### Noise Mitigation Techniques

#### RETROICOR

The at best modest improvements afforded by techniques used to reduce noise in this study suggest that common sources of physiological noise (cardiac pulsations and motion due to respiration) do not sufficiently explain the noise variance in the midbrain. When RETROICOR was applied to the high TR PRESTO and EPI scans, it only marginally reduced the influence of respiration and did not reduce noise in the cardiac frequencies. RETROICOR performed better for 2-D EPI scans compared to 3-D PRESTO scans, which corroborates results of our previous 7 T cortical fMRI analyses [66]. However, even though the algorithm was shown to regress out some respiratory or cardiac fluctuations in 2-D single-shot EPI data, we did not observe a significant improvement in the temporal stability of the data (Figure 1). This suggests that there are other noise sources influencing TSNR in the midbrain at 7 T.

#### Brainstem motion

From the retrospective brainstem cardiac-gated scan, we observed that the overall displacement of the midbrain was maximal in the anterior and inferior directions (0.05 mm in the anterior direction and 0.11 mm in the inferior direction, Figure 2). When considering a spatial resolution of 1.33 mm×1.33 mm×1.33 mm (∼2.35 mm^3^ volume), these maximal displacements still represent less than 10% of the voxel volume. It therefore seems unlikely that bulk motion associated with cardiac pulsatility is a significant source of temporal variance in the midbrain, consistent with the fact that the application of RETROICOR has little effect on midbrain TSNR.

#### Phase regression

The phase regression analysis was implemented in an attempt to reduce extraneous variance in the magnitude time course of individual voxels that would affect BOLD signal detectability. This approach, nevertheless, did not improve average TSNR in the midbrain (Figure 1). Moreover, the midbrain is densely vascularized with arteries and veins running through the SN and VTA in an oriented fashion. Some of the vessels running through the midbrain ROIs are greater than 25 µm in radius, risking the chance that BOLD activity in these regions will be regressed out by the algorithm. Hence, the phase regression technique may suppress BOLD signal changes in the SN and VTA as well as other sources of extraneous temporal variance. Within the constraints of this technique, we determined that temporally correlated changes in magnitude and phase (resulting from susceptibility variations associated with blood flow changes or B_0_ field inhomogeneities) do not explain the high temporal variance in the midbrain.

#### Power spectrum maps

High temporal resolution PRESTO and EPI sequences were used to create power spectrum maps that showed the spatial distribution of different frequency components in the midbrain (Figure 3). Resting state functional data acquired with PRESTO displayed variations in the cardiac frequency range concentrated around the midbrain area, possibly contributed by CSF pulsatility in the IPF and the arteries surrounding the lateral edges of the midbrain. However, high power values at cardiac frequencies did not propagate into the adjacent midbrain areas (SN and VTA). This indicates that noise in the midbrain ROIs cannot be explained by the cardiac noise. EPI on the other hand, demonstrated greater noise in the respiratory frequency range around the midbrain in comparison to the cardiac frequency. Here again the spatial distribution of the noise did not propagate into the midbrain regions, suggesting that respiration is not a dominant noise source in the midbrain DA nuclei. Thus, analyses of noise power spectra suggest that the dominant source of the noise in the midbrain cannot be attributed to either cardiac or respiratory fluctuations.

#### 1-D navigator approach

One of the disadvantages of multi-shot sequences is the accumulation of different phase errors between shots because k-space is acquired in segments separated by tens or hundreds of milliseconds. In the reconstruction of multi-shot data, phase shifts between shots produce image artifacts or ghosts that can spatially overlap with a region of interest. These ghosts can also vary over time, producing additional temporal variance. A 1-D navigator echo was implemented to mitigate this problem while imaging the midbrain using PRESTO and FFE sequences. The application of 1-D navigator correction on resting state FFE and PRESTO data demonstrated a TSNR improvement in the cortex, but very little improvement in the midbrain (Figure 4). A 1-D navigator is not optimal because it cannot fully account for spatially varying magnetic field inhomogeneities, but it is likely preferable to using only a global phase correction or no correction at all [51]. A 2-D phase correction algorithm may be used in future studies to produce more accurate estimates of phase changes throughout multi-shot acquisitions [67]. Thus, within the constraints of this algorithm, we established that phase offsets between shots do not sufficiently explain the observed high temporal variance in the midbrain.

#### Regressor of no interest analysis

We extracted signals from ROIs (white matter and anterior cerebellum) that were not functionally correlated with the midbrain SN and VTA. Only low temporal correlations were observed between the midbrain area and these non-interest regions, and so regression analyses did not significantly improve TSNR in the SN or VTA (Figure 5). Thus, areas near the midbrain regions of interest are not affected by the same source of variation, which rules out motion or other global effects as possible origins of noise in the midbrain.

#### Variations associated with functional connectivity

We observed that the mean MR signal in the midbrain was comparable to the cortex. However, the TSNR in the cortex was double or triple that of the midbrain due to large temporal variations in the midbrain. We postulated that the high temporal variance might be driven by intrinsic fluctuations between homologous midbrain regions. Partial correlation values observed between the left and right SN, and the left and the right VTA in the band-pass filtered 3-D FFE and the 3-D PRESTO scans demonstrated such a trend (Figure 6). The 2-D EPI results did not demonstrate the same trend as the 3-D scans, which may be explained by the fact that the EPI images also suffered more from distortions caused by susceptibility variations through space and time, as well as blurring from T2* effects [68].

ROI seed-based functional connectivity analyses between structures in the midbrain showed inter-regional correlations (Figure 7) that were observed in both 3-D sequences across individuals. The strongest associations were detected between the left and the right homologous ROIs, which we would expect to demonstrate the strongest functional interactions.

### Conclusions

Functional imaging of small midbrain structures is challenging, and midbrain fMRI studies at 3 T have relied upon large group analyses to overcome low BOLD contrast and/or high noise to achieve conclusive results [69]. We have attempted to exploit increased BOLD contrast at 7 T to perform single-subject fMRI analyses of midbrain dopaminergic regions. Unfortunately, at 7 T we observed consistently low TSNR in the midbrain while simultaneously achieving high TSNR in cortical regions. This unexpected observation prompted an investigation into several possible explanations for this low TSNR, and an evaluation of different acquisition and post-processing strategies to increase or otherwise restore midbrain TSNR to a level that would be useful for single-subject analyses. We observed that physiological fluctuations associated with cardiac pulsatility and respiration, phase instabilities between shots in 3-D multi-shot sequences, and the shared variance with temporally (but not functionally) correlated regions did not prominently influence noise in the midbrain. An evaluation of signal and noise in the midbrain and visual cortex revealed comparable mean signal levels but considerably higher temporal variance within the midbrain compared to the cortex. Our ROI seed-based functional connectivity analyses revealed strong intrinsic temporal BOLD fluctuations that appear to contribute to the relatively large variance in signals from the dopaminergic regions of the midbrain. These findings suggest that future work should continue to explore and characterize sources of physiological noise and low-frequency resting state fluctuations in midbrain structures. Potentially the apparent low values of TSNR in the midbrain actually reflect increased temporal variations that contain information on neural activity.
